# Pelvic Fractures in Adults and the Importance of Associated Injuries—A Current Multi-Disciplinary Approach

**DOI:** 10.3390/clinpract15070130

**Published:** 2025-07-11

**Authors:** Jakub Ohla, Piotr Walus, Michał Wiciński, Bartłomiej Małkowski, Bartosz Turoń, Adam Jabłoński, Michał Gawryjołek, Katie Kellett, Jan Zabrzyński

**Affiliations:** 1 Department of Orthopaedics and Traumatology, Faculty of Medicine, Collegium Medicum in Bydgoszcz, Nicolaus Copernicus University in Toruń, 85-092 Bydgoszcz, Poland; jakub.ohla@wp.pl (J.O.); jan.zabrzynski@cm.umk.pl (J.Z.); 2 Department of Orthopaedics and Traumatology, Faculty of Medicine, Jan Kochanowski University in Kielce, 25-001 Kielce, Poland; 3 Department of Pharmacology and Therapeutics, Faculty of Medicine, Collegium Medicum in Bydgoszcz, Nicolaus Copernicus University in Toruń, 85-092 Bydgoszcz, Poland; 4 Department of Urology, Oncology Centre Prof. Franciszek Łukaszczyk Memorial Hospital, Bydgoszcz, 85-796 Bydgoszcz, Poland; 5 Department of Orthopaedics and Traumatology, Władysław Biegański Regional Specialist Hospital, 86-300 Grudziądz, Poland

**Keywords:** pelvic ring injury, acetabular fracture, polytrauma, vascular injury, genitourinary injury

## Abstract

Background: Pelvic ring fractures are a significant and growing health problem in the field of trauma and orthopedic surgery. The aim of this paper was to present a concise description of these musculoskeletal injuries, available classification systems, and vascular and genitourinary complications. Results: The most common complications of serious pelvic ring fractures are arterial and venous hemorrhages, as well as urethral injuries. Arterial hemorrhages most often originate from the trunk or branches of the iliac artery, and the standard treatment is pelvic stabilization and implementation of intravascular procedures. In the case of venous hemorrhages, peritoneal pelvic packing is the most important. Conclusions: A multi-disciplinary approach and treatment algorithmization are important to facilitate the prioritization of therapeutic procedures. Treatment of patients with pelvic ring fractures should take place in specialized trauma centers.

## 1. Introduction

### 1.1. General Characteristics and Epidemiology

High-energy fractures of the pelvic ring and hip acetabulum are a serious medical problem because they pose a significant burden to healthcare systems [1,2,3]. They require long-term and interdisciplinary medical care and may result in dysfunction of the musculoskeletal system, especially gait disorders [4,5]. Surgical treatment of these fractures is carried out in qualified centers with trained medical staff and appropriate equipment [4,5,6].

Statistical data regarding pelvic fractures have not been clearly established. Population studies indicate that the average incidence of pelvic fractures is 20 per 100,000 people. The main mechanisms of injury are traffic accidents, injuries to pedestrians and cyclists as a result of being hit by vehicles, and falls from heights [7]. In 2021, in one of the literature reviews based on fifteen articles, it was shown that the main mechanism of injury in the case of open pelvic fractures are traffic accidents, which account for 67.1% of injuries [8]. High-energy pelvic injuries usually affect young men [7] and the majority of patients with open pelvic fractures are men (74.9%) with an average age of 35.1 years [8]. According to trauma registries in different countries, unstable pelvic fractures are associated with a mortality rate of 8 to 32%. Due to the increasing standard of care for trauma patients, there is a trend towards reduced mortality in patients with severe pelvic fractures; however, despite medical advances in this field, in the subgroup of patients with pelvic fractures complicated by hemorrhagic shock, the mortality rate is reported to be 32% [3]. The reason for this high mortality rate is that multi-focal hemorrhage can occur in the pelvis and cannot be easily stopped or controlled by traditional surgical methods such as ligation of a blood vessel or removal of an organ. Injury to the complex venous plexuses and, less commonly, arterial bleeding of disrupted iliac arteries are the most common causes of pelvic hemorrhage [9,10,11]. Treatment of this hemorrhage requires repositioning of the bony structures [12]. Severe pelvic injuries may be accompanied by concomitant injuries affecting the head, chest, abdominal organs, pelvis, and urogenital system [13]. Low-energy trauma can also lead to pelvic fractures, especially in older people [1]. Such injuries are often simple falls and are often associated with severe osteoporosis and usually affect women [14,15,16]. Despite the fact that over 70% of pelvic fractures are caused by low-energy trauma, the majority of them are stable fractures that do not require surgical interventions [5]. However, given that most of the affected individuals have comorbidities, such as chronic heart failure or anemia, a routine hemoglobin control within the first few days should be obtained in order to prevent from excessive blood loss [5,11]. A thorough examination, also during follow-up visits, is mandatory in order to avoid missing an unstable fracture, such as pubic rami and associated sacroiliac joint injury. Though the rate of misdiagnosis has not been defined, it has been reported and advised to expand the diagnostic to CT scan if the pain is persistent and does not resolve with time [17,18].

### 1.2. Aim and Study Design

In order to present the current concepts of the multi-disciplinary approach in pelvic fractures in adults, the authors decided to study recent papers and recommendations regarding this issue. To put the obtained data into perspective, a general outline of pelvic injuries and common classifications are presented before further analysis. The aim of this study was to present a concise description of these musculoskeletal injuries, available classification systems, and vascular and genitourinary complications.

## 2. Classifications of Pelvic Fractures

There are many classifications of pelvic fractures [6]. The most commonly used classification is the Young and Burgess classification (YBC). The original YBC is based on the mechanism of injury, and three main groups are distinguished based on the action of the main force vector causing the injury [19]. Within this classification, the authors distinguished the anterior–posterior compression (APC), lateral compression (LC) and vertical shear (VS) types [20]. YBC has been updated based on further research and integrated with the AO classification system. A comparison of both classifications is given in Table 1 [21]. Both intra- and interobserver reliability for this classification are satisfactory, but their values depend on the experience of the observer [22]. There is some debate as to whether the YBC is an adequate system for classifying pelvic ring injuries in children. Some authors indicate that there is a need to establish a separate classification of pelvic fractures in children, assessing in particular their stability and, on this basis, indicating the need for surgical treatment, which may improve the functional results of these fractures in this group of patients [23]. In the opinion of Igboechi et al., YBC, and the Arbeitsgemeinschaft für Osteosynthesefragen/Orthopedic Trauma Association (AO/OTA), these classifications are considered to adequately determine the severity of pelvic fractures in children. They also allow for the implementation of appropriate procedures, although they do not fully describe possible fracture patterns in this group of patients [24]. The same authors point out that Torode and Zieg’s (TZC) classification is also considered important. This classification divides pediatric pelvic fractures into four types (avulsion fracture, fracture of the iliac wing, fracture of the ring with no segmental instability, and fracture of the ring with segmental instability). Mennen et al. reviewed the literature and found eight articles in which the TZC was used. Based on their analysis, the authors concluded that the TZC is used relatively often to assess fracture stability, even though this system does not actually assess mechanical instability. Moreover, the authors believe that the YBC remains the optimal classification system and that it is important to create a pediatric modification of the YBC for the assessment of mechanical instability of pelvic fractures, allowing for better treatment planning [23]. Pelvic fractures in the geriatric group of patients have separate characteristics, therefore a separate classification system according to Rommens and Hofmann was proposed for low-energy and fatigue fractures [25]. Rommens’ classification is known as the Fragility Fractures of the Pelvis (FFP) classification [26]. There are four types of fractures with subtypes within FFP, and the classification criteria are fracture stability and its location [27]. The advantage of the classification is the assessment of fracture stability [26], which in turn allows for the assessment of indications for surgical treatment [28]. The FFP classification is characterized by moderate intra-rater and inter-rater reliability [17]. It is believed that in the case of fractures in geriatric patients, the classification of patients should be based on CT and MRI examination, which makes the classification system more objective [29]. An argument for performing extended diagnostic imaging using MRI is the progression of the fracture over time and its long-term course [30]. Data on the distribution of specific types of pelvic injuries are unclear. One of the retrospective studies assessed the incidence of particular types of fractures according to the OTA/AO classification. In a group of 187 patients with pelvic fractures older than 15 years, it was found that the most common type of pelvic fracture was type A (54.5%), followed by types B (36.9%) and C (8.6%) [31].

## 3. Pelvic Fractures and Associated Injuries

The characteristics of pelvic injuries mean that some patients require care from an interdisciplinary team of specialists due to accompanying injuries to the organs of the chest, abdominal cavity, and pelvis, as well as to neurovascular structures. Based on one of the retrospective studies involving 135 patients, no characteristic patterns of injuries were identified, understood as a correlation between a specific type in the YBC and concomitant injuries requiring urgent surgical intervention [32]. A correlation of pelvic fractures with other injuries was developed based on Tile’s classification (Table 2). In type A according to Tile, pelvic fractures were associated with fractures of the scapula, and in type B with fractures of the sacrum and injuries of internal organs [13]. Based on a clinical trial involving 16,000 patients, it has been shown that pelvic fractures are sometimes associated with intra-abdominal lesions, with an incidence of up to approximately 20%. Patients with concomitant abdominal trauma are younger and, in this group of patients, the mortality rate is significantly higher [33].

### 3.1. Vascular Injuries

Vascular injury and hemorrhage are the causes of relatively high mortality in pelvic fractures [34], and active arterial bleeding accompanying a pelvic fracture is most often the result of damage to the trunk or branches of the internal iliac artery [35]. Injury to the main iliac arteries, common, internal, and external are usually fatal and cause an immediate death due to the massive arterial bleeding [36,37]. Hemodynamically unstable patients that survived the initial few hours following pelvic fracture usually sustained an injury to the branches of the main iliac vessels and venous plexuses within the pelvic cavity and sacral bone [38]. In a retrospective study of 127 patients with high-energy pelvic ring fractures, vascular injury was found in 11.8% of cases. The most common vessels injured were the obturator, superior gluteal, and inferior gluteal arteries, respectively [39]. Based on a retrospective analysis of 2042 cases of pelvic fractures, the incidence of injury to the superior gluteal artery (SGA) was estimated at 1.5%. The mechanism of injury to this vessel and the patterns of pelvic fractures that predispose to it are unknown and further studies concerning this issue are strongly needed. Injury to the SGA may also occur in the absence of bony injuries in the sciatic notch [40]. In the study by Kim et al., based on the analysis of a similar study group, it was shown that of 148 patients, 28 (18.9%) showed pelvic arterial bleeding after blunt trauma. This bleeding was confirmed by enhanced computed tomography or angiography or by intraoperative assessment [41]. In another study performed by Kim et al., urgent surgical interventions to control hemorrhage were most frequently performed in patients with type B fractures (54.2%), according to the OTA/AO classification [31]. In a multi-center study by Constantini et al., which evaluated a group of 163 patients with pelvic fractures in hemorrhagic shock, it was shown that 83% of patients with APC III fractures required urgent surgery to control the hemorrhage. The mortality rate of patients with a pelvic fracture in hemorrhagic shock was 30% and was not dependent on the type of pelvic fracture [42]. Based on a retrospective analysis of 576 patients with high-energy pelvic fractures, it was found that post-traumatic hemorrhage extended beyond the pelvis to the gluteal region in 81% of cases, then to the abdominal area in 47%, and to the thighs in 25% [43]. Venous bleeding accounts for approximately 80% of cases of active bleeding following pelvic fractures, with the most common sources being the presacral plexus and prevesical veins [44]. The treatment of choice in this case is peritoneal pelvic packing and mechanical pelvic ring fixation, while endovascular treatment is used in the case of active bleeding from the main trunk of the iliac vein [45]. In case of injury to large arterial vessels, the basic treatment of choice is transcatheter arterial embolization (TAE). In hemodynamically stable patients, embolization of a selective branch of the internal iliac artery is performed [35]. This procedure is associated with an increased risk of venous thromboembolism (VTE), therefore VTE prophylaxis should be implemented as soon as possible in patients who have undergone this procedure [46]. In general, the use of pharmacological thromboprophylaxis in isolated severe pelvic fractures reduces the risk of VTE and increases the survival rate [47]. Angioembolization seems to be the treatment of choice, even in the case of acute retroperitoneal hemorrhage [48]. It should be pointed out that the basic method of treatment in hemodynamically unstable patients is circumferential pelvic antishock sheeting, and the effectiveness of this method results from reducing the pelvic volume. It is important to use proper techniques to determine effectiveness, ability to perform resuscitation, and access to the abdomen and limbs [49].

### 3.2. Genitourinary System Injuries

In a large study group consisting of over 180,000 cases, it was shown that pelvic fractures with accompanying injuries of the lower gastrointestinal tract and structures of the urogenital system constitute <1% of all fractures. The strongest risk factors for such injuries are disruption of the pelvic ring, innominate bone fracture, and male gender [50]. Injuries to the bladder and urethra are more common in injuries to the anterior part of the pelvic ring. These injuries may cause permanent dysfunction of the urogenital system [51]. In one of the meta-analyses, which included eight studies with a total of 343 patients, the incidence of urogenital trauma associated with pelvic fractures was estimated at 19.7%, with the authors reporting that 32.4% of women suffered vaginal lacerations [52]. Kaneko et al. performed a retrospective analysis of 402 patients with pelvic fractures. Urinary and genital injuries were found in 18 of them, which constituted only 4.5% of the study group. The group of patients with pelvic fractures and concomitant genitourinary injuries were statistically significantly younger and more frequently required hospitalization in the intensive care unit [53]. Pelvic fracture urethral injury (PFUI) is an important issue. PFUI has a wide spectrum of severity. Both minor and major urethral injuries can result in its obstruction and the need for urethroplasty and urethral obstruction following partial PFUI is observed in 50% of cases [54]. In patients with concomitant pelvic ring and bladder injuries, ORIF of anterior pelvic ring fractures is not associated with an increased rate of infection [55]. However, operative treatment of pelvic fractures with concomitant urethral injury using implants in the anterior part of the pelvic ring is associated with an increased risk of infection, but this risk is not increased in fractures with extraperitoneal injury to the lower urinary tract [56]. There are few studies assessing late mortality caused by inflammatory complications. The mainstay of treatment for such infections is parenteral and local antibiotic therapy [52].

## 4. Conclusions

Due to the development of medicine, including the emergency medical system, as well as diagnostic and treatment methods in the field of emergency medicine and intensive care, the quality of pre-hospital care and the survival rate of patients with severe multi-organ injuries are increasing. In trauma centers and reference centers for trauma treatment organized within the healthcare system, the presence of multi-disciplinary trauma teams focused on the treatment of multi-organ injuries becomes important. It seems crucial to create a far-reaching algorithmization of diagnostic and therapeutic processes allowing for prioritization of treatment, as well as cooperation between individual members of the trauma team aimed at building a treatment plan that optimizes the therapeutic process.

## Figures and Tables

**Table 1:** Two major classifications of pelvic fractures.

AO/OTA Classification			Young–Burgess Classification		
Description		Type	Morphological Description of the Injury		Force Applied
Lesion sparing (or no displacement of) posterior arch; STABLE.		A	Symphyseal diastasis or longitudinal rami fractures.		Antero-posterior Compression (APC)
			Symphysis widening < 2.5 cm.	Type I	
			Symphysis widening > 2.5 cm. Anterior sacroiliac (SI) joint diastasis. Posterior SI ligaments intact. Disruption of sacrospinous and sacrotuberous ligaments.	Type II	
			Disruption of anterior and posterior SI ligaments (SI dislocation). Disruption of sacrospinous and sacrotuberous ligaments.	Type III	
Incomplete disruption of posterior arch; PARTIALLY STABLE.		B	Transverse fracture of pubic rami, ipsilateral, or contralateral to posterior injury.		Lateral Compression (LC)
Unilateral, partial disruption of posterior arch, external rotation.	B1		Oblique or transverse ramus fracture and ipsilateral anterior sacral ala compression fracture.	Type I	
Unilateral, partial disruption of posterior arch, internal rotation. B2.1 Anterior compression fracture of sacrum. B2.2 Partial sacroiliac joint fracture/subluxation. B2.3 Incomplete posterior iliac fracture.	B2		Rami fracture and ipsilateral posterior ilium fracture dislocation (crescent fracture).	Type II	
Bilateral, partial disruption of posterior arch. B3.1 Bilateral B1, B3.2 B1 + B2. B3.3 Bilateral B2.	B3		Ipsilateral lateral compression + contralateral open-book fracture (APC III).	Type III	
Complete disruption of posterior arch; UNSTABLE.		C	Symphyseal diastasis or vertical displacement in anterior or posterior directions, typically through SI joint, but occasionally through the sacrum or iliac wing.		Vertical Shear (VS)
			Combined injury pattern, with LC/VS being the most frequent.		Combined Mechanism of Injury (CMI)

**Table 2:** Tile classification of pelvic fracture.

Type A	Type B	Type C
Stable (posterior arch intact)	Partially stable (incomplete disruption of the posterior arch)	Unstable (complete disruption of the posterior arch)
A1: Avulsion injury	B1: Open-book injury (external rotation)	C1: Unilateral • C1-1: Iliac fracture • C1-2: Sacroiliac fracture-dislocation • C1-3: Sacral fracture
A2: Iliac-wing or anterior-arch fracture due to a direct blow	B2: Lateral-compression injury (internal rotation) • B2-1: Ipsilateral anterior and posterior injuries • B2-2: Contralateral (bucket-handle) injuries	• C2: Bilateral, with one side type B, one side type C
A3: Transverse sacrococcygeal fracture	B3: Bilateral	• C3: Bilateral

## Data Availability

The data presented in this study are available on reasonable request from the corresponding author.
